# Caregiver Spouses of Persons with Dementia who Engage with an Emergency Preparedness Toolkit Intervention

**DOI:** 10.1093/geroni/igaf122.2351

**Published:** 2025-12-31

**Authors:** Anna Satake, Rebecca Boxer, Andrea Daddato, Kris Wain, Blythe Dollar, James Lagrotteria

**Affiliations:** University of California, Davis, Davis, California, United States; University of California, Davis, Davis, California, United States; Kaiser Permanente Colorado, Aurora, Colorado, United States; Kaiser Permanente Colorado, Aurora, Colorado, United States; Kaiser Permanente Colorado, Aurora, Colorado, United States; Kaiser Permanente Colorado, Aurora, Colorado, United States

## Abstract

Hospitalizations of a spousal caregiver (SC) of a person with dementia (PWD) can significantly disrupt caregiving and negatively impact the SC. This study conducted a low-touch intervention by offering a SC an emergency preparedness toolkit (EPT) to prepare for their own unexpected hospitalization. In January 2023, 1,062 caregivers of PWD who live together and are not institutionalized were invited to request the EPT and use it to prepare for their own healthcare emergency. Of caregivers notified, 489(46%) requested the EPT. Caregivers who didn’t request the EPT n = 573(54%) were more likely to be male [261(46%) vs 188(38%);p=.02], have higher Elixhauser comorbidity scores (3.0±2.6 vs 2.5±1.9, p<.01), have more ED visits[132(23%) vs 88(18%)p=.04], and hospitalizations [77(13%) vs. 34(7%);p = <.01] in the prior 12 months compared to caregivers who requested the EPT. Caregiver outcomes by EPT request were measured 6-month after the EPT was sent. Caregivers who didn’t request the EPT were no different in number of hospitalizations [52(9% vs 34(7%), p=.2] than those who did; however, they had a higher number of deaths [13(2%) vs 2(.4%), p=.01] at 6 months. For the caregiver, there were no significant differences in outcomes such as ED visits/hospitalizations between the two groups. Findings indicate that the health severity of caregivers of PWD was associated with the caregiver’s response to a supportive intervention. Outreach and engagement to caregivers may need to be tailored to a range of caregiver health conditions and severity to be successful. Health of caregivers and their uptake of support and resources warrants further study.

